# Prognostic markers of post-stroke depression (PROMoSD): study protocol of a prospective single-center observational study on raphe hypoechogenicity as a predictor of post-stroke depression

**DOI:** 10.1186/s42466-022-00225-5

**Published:** 2022-12-09

**Authors:** Daniel Richter, Andreas Ebert, Lisa Mazul-Wach, Quirin Ruland, Jeyanthan Charles-James, Ralf Gold, Georgios Tsivgoulis, Georg Juckel, Christos Krogias

**Affiliations:** 1 Department of Neurology, St. Josef-Hospital Bochum, Ruhr University Bochum, Gudrunstr. 56, 44791 Bochum, Germany; 2 Department of Psychiatry, Psychotherapy and Preventive Medicine, LWL University Hospital, Ruhr University Bochum, Bochum, Germany; 3 Medical Faculty, Ruhr University Bochum, Bochum, Germany; 4 2nd Department of Neurology, National and Kapodistrian University of Athens, Attikon University Hospital, Athens, Greece

**Keywords:** Stroke, Post-stroke depression, Transcranial sonography

## Abstract

**Introduction:**

Post-stroke depression (PSD) is an important and frequent non-motor complication after a stroke. As valid prediction of PSD occurrence is still not possible, the unselective use of preventive therapy in stroke patients has risen a questionable risk-to-benefit consideration. Therefore, there is a need to increase the prediction probability of PSD to identify patients at very high risk of a depressive complication who might benefit from preventive therapy. In this context, a brainstem raphe hypoechogenicity (BRH) in transcranial sonography (TCS) has previously been associated with depressive symptoms in a broad spectrum of diseases. BRH might therefore represent a valid maker of vulnerability for depressive symptoms that could be of interest in the risk assessment of PSD occurrence.

**Methods:**

In the prognostic markers of post-stroke depression (PROMoSD) study, a prospective, observational, single-center, investigator-initiated study, we aim to include 100 patients with acute ischemic stroke (AIS). Besides data on clinical characteristics and baseline psychiatric assessment, we conduct a TCS examination to identify patients with BRH. The primary outcome is the incidence of PSD three months after inclusion, determined by a blinded investigator according to the fifth version of the Diagnostic and Statistical Manual of Mental Disorders (DSM-V) criteria.

**Perspective:**

The results of PROMoSD will answer the question of whether screening of BRH after AIS improves the prediction of PSD occurrence. A positive result of this study could have direct consequences on psychiatric support after AIS by streamlining diagnostic and therapeutic algorithms.

*Trial registration* ClinicalTrials.gov identifier no. NCT05580198.

## Introduction

Stroke represents one of the most common causes of disability and the need for care worldwide [1]. The consequences of a stroke are often severe and affect both motor and non-motor aspects. Post-stroke depression (PSD) is the most common complication after a stroke that contributes substantially to the impairment of the quality of life [2, 3].

Although preventive treatment approaches for PSD have been investigated, these trials have raised with moderate benefits but relevant side effects [4]. It has been postulated that the identification of risk factors for PSD could improve the risk-to-benefit consideration of preventive therapies. In the past, several unspecific PSD risk factors, including stroke severity, previous depressive episodes, and female sex, have emerged as the most common risk factors for developing PSD, but with overall low predictive values [5]. In other diseases, depressive symptoms have been associated with brainstem raphe hypoechogenicity (BRH) which is investigated by transcranial sonography (TCS) [6–10]. Previous data suggest that BR hypoechogenicity may be a general marker of vulnerability for depressive symptoms, but this has never been explored in a prospective study design [11].

## Methods

### Aim of the trial

There is a need for a better prediction of PSD occurrence. BRH is a potential marker of depression vulnerability. Therefore, the primary aim of the prospective, single-center observational study PROMoSD (prognostic markers of post-stroke-depression) is to investigate whether the presence of a BRH at baseline after an acute ischemic stroke (AIS) predicts the occurrence of a PSD. The secondary aim of this trial is to identify other risk factors that might be associated with an increased risk to develop a PSD three months after an AIS.

### Study description and design

PROMoSD is a prospective, observational, single-center, investigator-initiated study which is conducted in the western area of Germany. The local neurological teams of the Department of Neurology of the St. Josef-Hospital Bochum, Ruhr University Bochum, review patients upon admission and will assess eligibility for inclusion in the PROMoSD study. Before inclusion, patients need to give informed consent.

**Fig. 1:**
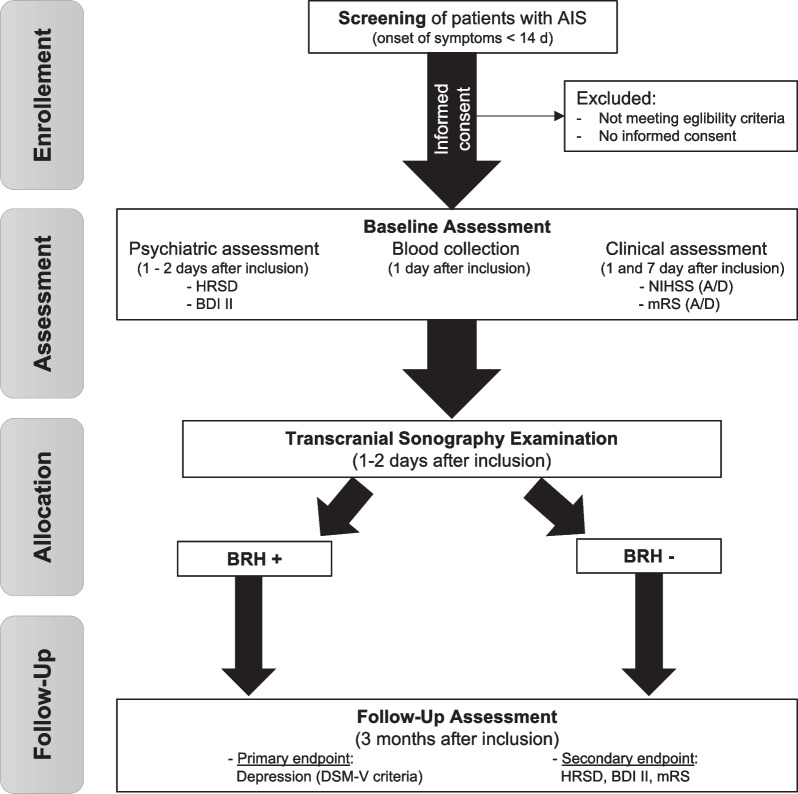
PROMoSD study flowchart. *AIS* acute ischemic stroke, *HRSD* Hamilton Rating Scale for Depression, *BDI II* Beck’s Depression Inventory II, *NIHSS* National Institute of Health Stroke Scale, *mRS* Modified Rankin Scale, *DSM-V* Fifth version of the Diagnostic and Statistical Manual of Mental Disorders

We hypothesize that the presence of a BRH leads to a significant increase in the incidence of PSD. The TCS examination to investigate the presence of a BRH is conducted after inclusion and by investigators who are blinded to the clinical information of the patients. Besides TCS, routine clinical information, common stroke severity scores, serum blood samples, and magnetic resonance imaging data of the brain are collected if available at baseline. The applied set of assessments covers self-reported outcome measures as well as objective scores questioning the presence and severity of depressive symptoms. Initial measures are conducted during the baseline hospital stay. The primary endpoint is the frequency of PSD three months after inclusion which is compared between patients with and without BRH. The Follow-up investigation takes place in a University Hospital for Psychiatry (Fig. 1). The initial and follow-up psychiatric assessment is conducted by blinded investigators. Data collection is carried out according to German data protection law.

### Arms and diagnostic intervention

After inclusion, the patients are stratified according to their brainstem raphe status which is determined by the TCS examination. TCS is performed by experienced and board certified (Deutsche Gesellschaft für Ultraschall in der Medizin, DEGUM) investigators who are blinded to any clinical information of the patients. On the other hand, clinical and psychiatric investigators have no access to the TCS data at any time during the study. TCS examination is conducted using a phased array ultrasound system equipped with a 2.5-MHz transducer (Aplio XG Ultrasound System, Toshiba Medicals, Tochigi, Japan). For technical configuration, a penetration depth of 150 mm and a dynamic range of 45–50 dB are chosen. Image brightness and time gain compensation are adapted as needed for each examination. The examination protocol is based on the previously published recommendations for TCS [12]. In detail, using the transtemporal approach, the midbrain and diencephalic examination planes are visualized in the axial section. The grade of BR echogenicity is assessed semi-quantitatively on a three-point scale (0 = raphe structure not visible, 1 = slight and interrupted echogenic raphe structure, 2 = normal echogenicity). In further analysis, grades 0 and 1 are pooled together as hypoechogenic (BRH+: interrupted or absent raphe structure) versus normoechogenic (BRH−: echogenicity of raphe structure is not interrupted and intensity is equal to that of the red nucleus, grade 2) [10]. The sonographic findings are stored to perform a second evaluation and classification of the results by a second investigator. In case of discrepant ratings, a consensus is accomplished.

### Outcome measures

The primary outcome is the presence of a PSD in the follow-up examination three months after inclusion. PSD is diagnosed by a blinded investigator from the Department of Psychiatry, Psychotherapy and Preventive Medicine, LWL University Hospital, Ruhr University Bochum, Germany. PSD diagnosis is defined according to the fifth version of the Diagnostic and Statistical Manual of Mental Disorders (DSM-V) criteria. Furthermore, patients who receive new medication for anti-depressive indications until follow-up are defined as PSD-positive.

Secondary outcomes are the severity of depressive symptoms measured by the Hamilton rating scale for depression (HRSD), the Beck’s Depression Inventory II (BDI II) as well as the functional outcome measured by the modified Rankin Scale (mRS). Primary and secondary outcomes are assessed at the same time point (Table 1).

**Table 1:** List of applied tests given for each time point

Time point	T0		T1
	T0a	T0b	
*Method*			
Beck’s Depression Inventory II (BDI II)	X		X
Hamilton Rating Scale for Depression (HRSD)	X		X
Blood collection	X		
Transcranial Sonography (TCS)	X		
National Institute of Health Stroke Scale (NIHSS)	X	X	
Modified Rankin Scale (mRS)	X	X	X
Post-Stroke Depression according to DSM-V criteria			X

T0: Baseline assessment (T0a: 1–2 days after inclusion, T0b: 7 days after inclusion), T1: Follow-up assessmen three months after inclusion

DSM-V, Diagnostic and Statistical Manual of Mental Disorders

### Eligibility criteria

Patients admitted to the cooperating Stroke Center following acute ischemic stroke are screened by a physician for eligibility.

The inclusion criteria are:

- Patients with ischemic stroke confirmed by brain imaging (either computer tomography or magnetic resonance imaging)
- Onset of AIS symptoms within the past 14 days
- Age ≥ 18 years
- Sufficient German language skills
- Declaration of consent

The exclusion criteria are:

- No transtemporal bone window for TCS examination

### Sample size estimation

We determined that a sample of 100 patients would provide the study with a power of 80% to detect an absolute relative difference of 30% in PSD occurrence with a two-sided alpha level of 0.05 based on the assumption of a 20% prevalence rate of BR hypoechogenicity in a non-depressed population in Germany.

### Contacts

The study was initiated by the Department of Neurology of the St. Josef-Hospital Bochum in cooperation with the Department of Psychiatry, Psychotherapy and Preventive Medicine of the LWL University Hospital Bochum. The study is funded by the Ruhr University Bochum (FoRUM grant K136-20). The study was approved by the local University ethics committee of the Ruhr University Bochum, Germany (approval no. 20-6862). To ensure compliance with the rules of good clinical practice, this study is being monitored by a steering committee (Prof. Christos Krogias [Chair], Department of Neurology, St. Josef-Hospital Bochum, Ruhr University Bochum, Germany; Prof. Georg Juckel, Department of Psychiatry, Psychotherapy and Preventive Medicine, LWL University Hospital, Ruhr University Bochum, Germany; Prof. Georgios Tsivgoulis, Second Department of Neurology, School of Medicine, 'Attikon' University Hospital, National and Kapodistrian University of Athens, Greece, and Department of Neurology, The University of Tennessee Health Science Center, Memphis, TN, USA; Prof. Uwe Walter, Department of Neurology, Rostock University, Germany; Prof. Aristeidis Katsanos, Division of Neurology, McMaster University/Population Health Research Institute, Hamilton, Ontario, Canada).

## Perspective

PSD is probably the most important non-motor complication after a stroke with limited prediction accuracy [13]. Therefore, the PROMoSD study aims to improve PSD prediction by screening for BRH early after an AIS. Current evidence suggests that a BRH investigated by TCS is associated with depressive symptoms and might be a marker of vulnerability for depressive disorders. TCS by its nature is a non-invasive, cost-effective, and feasible diagnostic tool with broad availability and bedside opportunity which makes it very interesting for a wide application. A positive result of this study could have direct consequences for psychiatric support after AIS. Furthermore, it could create the basis for preventive BRH-related therapy trials with the possibility to streamline therapeutic algorithms.

## Data Availability

The datasets used and/or analyzed during the current study are available from the corresponding author upon reasonable request.

## Abbreviations

AIS: Acute ischemic stroke
PSD: Post-stroke depression
TCS: Transcranial sonography
BRH: Brainstem raphe hypoechogenicity
DSM-V: Fifth version of the Diagnostic and Statistical Manual of Mental Disorders
HRSD: Hamilton rating scale for depression
BDI II: Beck’s Depression Inventory II
mRS: Modified Rankin Scale
